# Correlation of procalcitonin and C-reactive protein to inflammation, complications, and outcome during the intensive care unit course of multiple-trauma patients

**DOI:** 10.1186/cc3910

**Published:** 2005-11-24

**Authors:** Michael Meisner, Heide Adina, Joachim Schmidt

**Affiliations:** 1 Department of Anaesthesiology and Intensive Care Medicine, Hospital Dresden Neustadt, Industriestrasse 40, D-01129 Dresden, Germany; 2 Department of Anaesthesiology and Intensive Care Therapy, Friedrich Schiller University Jena, Erlanger Allee 101, D-07740 Jena, Germany; 3 Department of Anaesthesiology and Intensive Care Therapy, University of Erlangen-Nuremberg, Krankenhausstrasse 12, D-91054 Erlangen, Germany

## Abstract

**Background:**

A comparison of the amount of and the kinetics of induction of procalcitonin (PCT) with that of C-reactive protein (CRP) during various types of and severities of multiple trauma, and their relation to trauma-related complications, was performed.

**Methods:**

Ninety adult trauma patients admitted to the intensive care unit of our tertiary care hospital were evaluated in a prospective case study. During the initial 24 hours after trauma the Injury Severity Score, the Sepsis-related Organ Failure Assessment score, and the Acute Physiology and Chronic Health Evaluation II score were evaluated. PCT, CRP, the sepsis criteria (American College of Chest Physicians/Society of Critical Care Medicine definitions), and the Sepsis-related Organ Failure Assessment score were measured at days 1–7, as well as at days 14 and 21, concluding the observation period with the 28-day survival.

**Results:**

The induction of PCT and CRP varied in patients suffering from trauma. PCT increased only moderately in most patients and peaked at day 1–2 after trauma, the concentrations rapidly declining thereafter. CRP ubiquitously increased and its kinetics were much slower. Complications such as sepsis, infection, blood transfusion, prolonged intensive care unit treatment, and poor outcome were more frequent in patients with initially high PCT (>1 ng/ml), whereas increases of CRP showed no positive correlation.

**Conclusion:**

In patients with multiple trauma due to an accident, the PCT level provides more information than the CRP level since only moderate amounts of PCT are induced, and higher concentrations correlate with more severe trauma and a higher frequency of various complications, including sepsis and infection. Most importantly, the moderate trauma-related increase of PCT and the rapidly declining concentrations provide a baseline value near to the normal range at an earlier time frame than for CRP, thus allowing a faster and more valid prediction of sepsis during the early period after trauma.

## Introduction

Multiple-trauma patients are especially prone to develop complications such as infections and sepsis. Since clinical symptoms and conventional markers are not always reliable signs for the diagnosis of sepsis and infection, biomarkers such as procalcitonin (PCT) or C-reactive protein (CRP) are often used as a diagnostic tool in these patients. Multiple-trauma patients, however, similar to patients undergoing elective surgery, may show an increase of PCT, CRP, and other biomolecules, indicating inflammation, during the early postoperative or post-traumatic period independent of the diagnosis of sepsis or infection [1-4].

Several studies previously described the kinetics and the amount of PCT induced after elective surgery and trauma [1,3-8]. The induction of PCT and CRP after surgery has been described quite well in the meantime: PCT levels increase far less than CRP levels, and the period of unspecific induction is much shorter [1,7]. The PCT parameter is therefore the better choice to diagnose sepsis and infection early after surgery. Data on CRP induction after multiple trauma are scarce, however, and provide no detailed data on the induction of this protein at various severity levels and types of trauma as compared with PCT [3,9].

The aim of this study was to describe the amount of and the time course of PCT and CRP induction in patients with various types of and severities of high-velocity trauma. We further registered trauma-related complications (for example, sepsis, infection, blood transfusion, organ dysfunction), as described by the Sepsis-related Organ Failure Assessment (SOFA) score, the Acute Physiology and Chronic Health Evaluation II (APACHE II) score, the duration of stay in the intensive care unit (ICU), and the overall outcome.

## Patients and methods

After approval by the local ethics committee, all patients with physical trauma due to an accident admitted to the ICU of our tertiary health care institution between May 1998 and April 2000 were prospectively included in the study. Inclusion criteria included age older than 16 years and survival for at least 12 hours. No chemical or burn trauma patients were included. Patients underwent surgical treatment when necessary for blood loss, wound treatment, or bone fractures according to accepted standards of care. PCT, CRP, all clinical, microbiological, and laboratory data, and all diagnostic and therapeutic options were registered. The data analyzed included data collected once during admission: age, gender, chronic conditions, severity of trauma according to the Injury Severity Score (ISS) [10], the APACHE II score [11], and number of blood products infused within the initial 24 hours after trauma. Also analyzed were data collected each day for 7 days, and on days 14 and 21 of treatment in the ICU: PCT, CRP, clinical evidence and laboratory data of infection, microbiological findings, clinical suspicion of infection, and the duration of treatment on the ICU, as well as the data necessary to evaluate the SOFA score [12]. Complications included the diagnosis of infection, systemic inflammation, the various stages of sepsis according to American College of Chest Physicians/Society of Critical Care Medicine criteria [13], and the occurrence of organ dysfunction. The final data analyzed were 28-day survival data.

The type of and severity of trauma was classified according to the ISS. Severe trauma was assumed at ISS ≥20 according to the consensus of previous publications [10,14]. Infection was diagnosed if microbiological cultures obtained from the patients at possible sites of infection were positive (proven infection) or if clinical signs of infection were evident. 'Suspected infection' was stated when the treating physician suspected a bacterial infection but no positive microbiological result was obtained (patients with proven infection are included in this group). Pneumonia was diagnosed if radiological signs of pneumonia (infiltration) on chest X-ray and at least one of the following two criteria were present: leukocytosis >12,000 × 10^9^/l or <4,000 × 10^9^/l, or body temperature >38°C or <36°C. Blood transfusions were given until the patient was hemodynamically stable or until hemoglobin values exceeded 8.0 g/dl, according to local guidelines.

The PCT level was measured by the Lumitest^®^PCT luminometric assay (B.R.A.H.M.S. AG, Berlin-Hennigsdorf, Germany) and the CRP level was measured using a nephelometric assay (Boehringer, Mannheim, Germany) [15]. The functional assay sensitivity of the Lumitest^®^PCT is 0.3 ng/ml. PCT concentrations were expressed as initial peak levels according to their maximum concentrations on day 1 or day 2 after trauma, and the CRP concentrations were from day 1 to day 3 after trauma due to the slower CRP induction kinetics.

The initial ISS categorizes the type of and severity of trauma according to a system of points based on injury to six regions of the body [10]. The ISS is defined as "the sum of the square of the highest points in each of the three most severely injured areas". The following regions are scored according to a scale of 0 (no injury) to 6 (major injury): head/neck, face, thorax, abdomen, extremities, and external wounds.

### Statistical analysis

Statistical evaluation was carried out using the program SPSS 10.0 for Windows. Variables were defined as the median and upper and lower quartiles. When Kruskal–Wallis analysis indicated a significant difference among groups, the Mann–Whitney *U *test was used to compare the groups. Correlations were calculated by the Spearman rank correlation. Increased risk was calculated by the odds ratio, and significance was tested by the chi-square test. The area under the curve of the receiver operating characteristic was calculated and plotted by SPSS 10.0. Statistical comparison between the area under the curve of the various parameters was calculated using the method developed by Hanley and McNeil [16], in which *z *> 1.96 indicates a level of significance or an alpha error less than 5%. The McNemar test was used for comparison of sensitivity and specificity among parameters and scores at a given cutoff point. Statistical significance was accepted for *P *< 0.05. A Bonferroni correction was calculated for each group of comparisons.

## Results

### Patient characteristics

Out of 102 patients with accidental high-velocity multiple trauma, 90 met the inclusion criteria during the study period. Twelve of the initially evaluated patients could not be followed up because of a fatal outcome within 12 hours. The median age was 34 years (range, 16–84 years; 29 female patients, 61 male patients). Eighty-four patients (93%) were traumatized due to motor vehicle accidents, whereas six patients (7%) suffered from trauma following a fall from a greater height. The ISS ranged between 5 and 50 (median, 24.5), and the duration of ICU treatment averaged 12 days (median, 8 days; range 1–28 days). Further baseline characteristics for the patients are presented in Table 1.

### Induction of PCT and CRP after trauma

**Figure 1:**
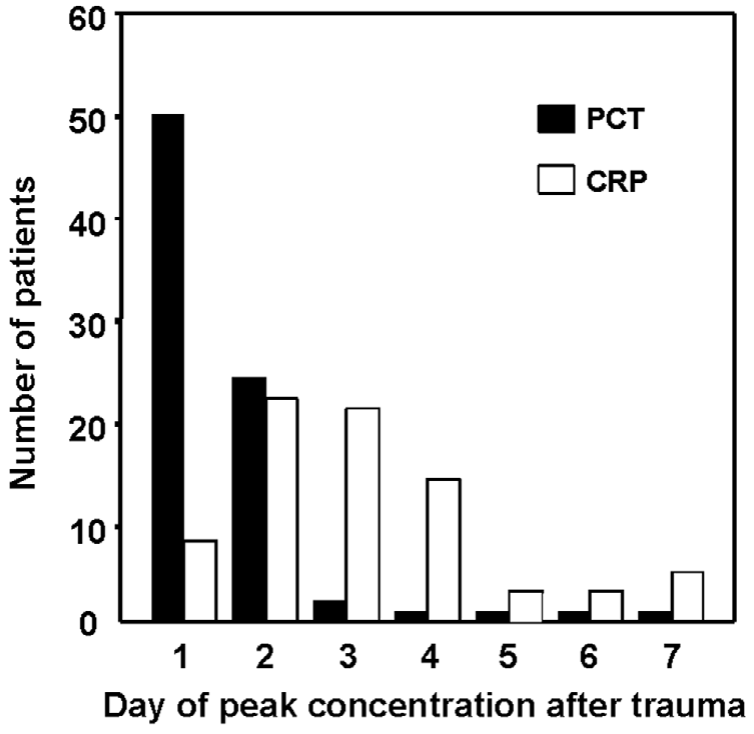
Histogram of the day of maximum concentrations of procalcitonin (PCT) and C-reactive protein (CRP) in multiple-trauma patients.

**Figure 2:**
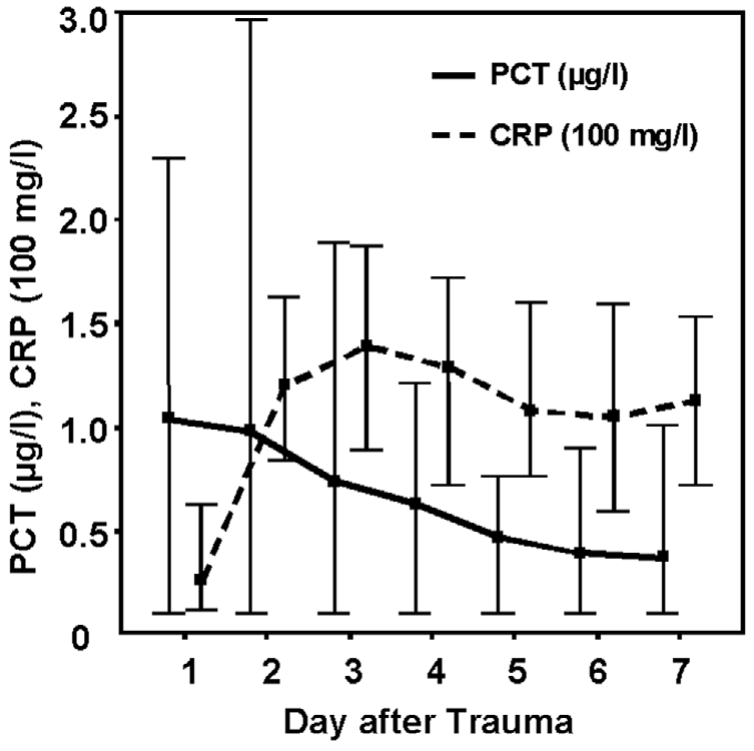
Serum levels of procalcitonin (PCT) and C-reactive protein (CRP) in patients with multiple trauma. Time course of induction (median, quartiles).

The PCT concentration was increased above the normal levels of 0.5 ng/ml in 71% of the patients on day 1 or day 2 after trauma. Peak concentrations occurred on day 1 in 56% of the patients and on day 2 in another 26% (Figure 1). The highest concentration of PCT measured was 18.7 ng/ml. In comparison, the time course of CRP induction was slower. Peak concentrations occurred only in 9% of the patients on day 1 after trauma, in 24% of the patients on day 2, and in 23% of the patients on day 3. Even on day 4 after trauma the peak levels of CRP were reached in another 15% of the patients. Initial CRP levels increased above 10 mg/l in 98% of patients, and 81 patients (90%) developed CRP levels above 50 mg/l. Concentrations did not exceed 365 mg/l during severe trauma. PCT concentrations declined more rapidly than those of CRP (Figure 2). On day 7 after trauma, the PCT level was within the normal range in 88% of the patients while the CRP level was within the normal range in only 6% of the patients.

### Influence of type of and severity of trauma

The majority of the patients presented with multiple trauma of various regions of the body. Seven patients were injured in only one or two regions, 25 patients in three regions, 35 patients in four regions, and 23 patients in five or more regions. PCT and CRP concentrations according to the region of injury are summarized in Table 2. There was no statistical difference in PCT levels between the specific trauma patterns; however, patients with abdominal trauma obviously presented with somewhat higher PCT levels (*P *= 0.004, corresponding to an adjusted alpha error of 6.5% for multiple comparisons). Eighty-five percent of the patients underwent surgical procedures during the initial observation period (days 1–2 after trauma). Initial PCT and CRP levels were similar in patients undergoing early surgery and those with no or late surgery. PCT concentrations, but not CRP levels, correlated with the number of blood units given on the initial day of trauma (*r *= 0.61, *P *< 0.001 and *r *= 0.20, *P *= 0.055, respectively). Accordingly, PCT concentrations were significantly lower in patients who had moderate blood loss (≤2 units blood transfused, PCT = 0.64 ng/ml) as compared with those with major blood loss (> 2 units transfused, PCT = 3.05 ng/ml, median; *P *< 0.001).

When patients were categorized into those with moderate or severe trauma (ISS <20 or ≥20), the initial PCT but not CRP was significantly higher in patients with severe trauma (*P *< 0.001 and *P <*0.177, respectively) (Table 1). Nevertheless, the initial PCT peak concentrations (day 1 or 2) correlated only weakly with the ISS (*r *= 0.416, *P *< 0.001), and the CRP concentrations (day 1–3) did not correlate at all with the ISS (*r *= 0.112, *P *= 0.295).

### Duration of ICU treatment and outcome

Patients with high PCT levels early after trauma were treated for a longer period of time in the ICU than those who initially presented with low PCT values. In the case of PCT levels greater than 2.5 ng/ml (day 1 or 2 after trauma) the average duration of treatment in the ICU was 17 (± 13) days, as compared with 5 (± 4) days in those with PCT < 0.5 ng/ml (*P *< 0.001). However, there was no distinct arithmetical correlation between both parameters (*r *= 0.500, *P *< 0.01).

**Figure 3:**
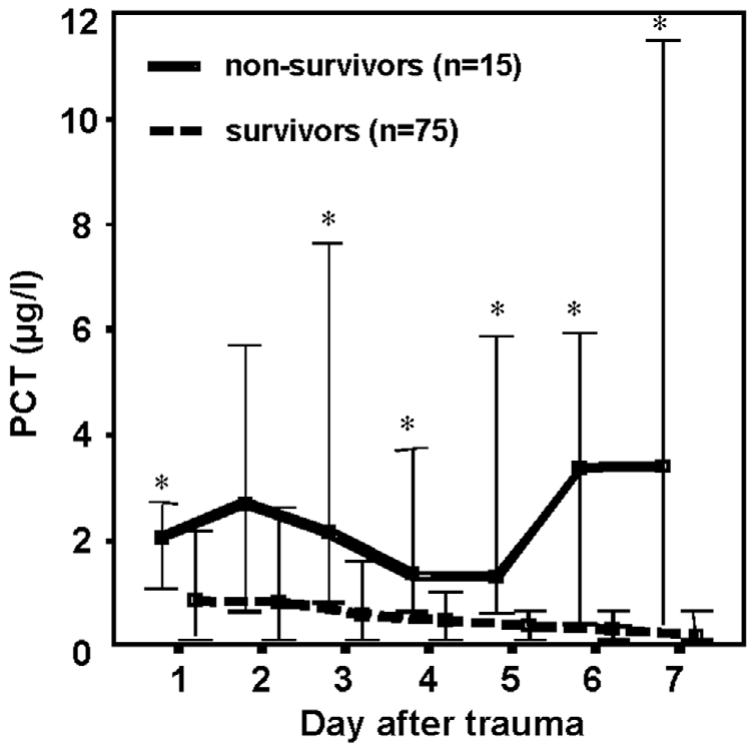
Course of procalcitonin (PCT) (median, quartiles) during the first week after trauma in nonsurvivors (*n *= 15) and in survivors (*n *= 75). *P < 0.05, Mann–Whitney *U *test.

**Figure 4:**
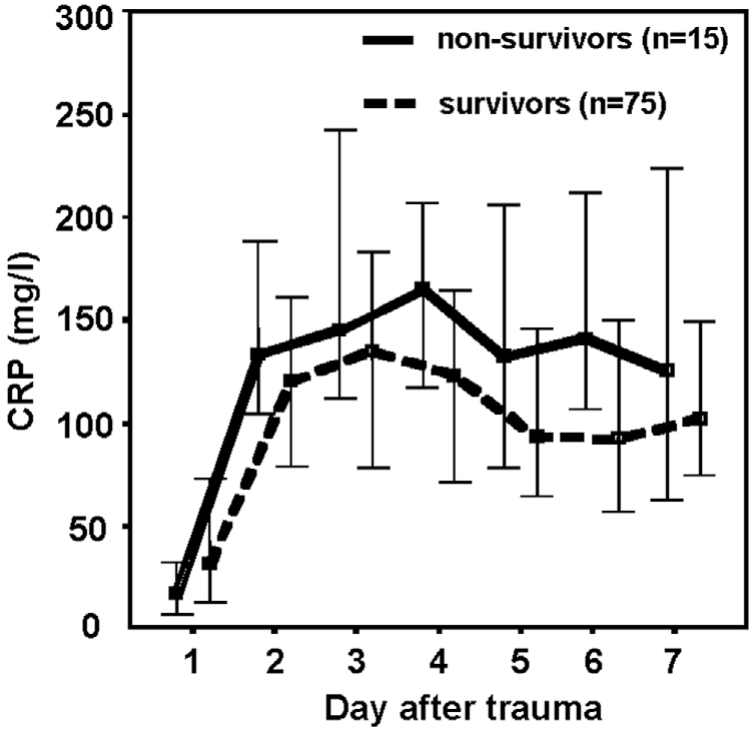
Course of C-reactive protein (CRP) (median, quartiles) during the first week after trauma in nonsurvivors (*n *= 15) and in survivors (*n *= 75).

Fifteen of the 90 patients analyzed died within 28 days after trauma, all following severe trauma according to an ISS of 27–50 (median, 41). The PCT, but not CRP, concentrations during the first week after trauma were significantly higher in nonsurvivors as compared with those in survivors (Figures 3 and 4). During the course of treatment, the difference among groups at the end of the first week even increased from double to 15-fold in patients with fatal outcome compared with in survivors. At a cutoff value of 0.8 ng/ml for the initial PCT concentration, the probability of survival was 94% (negative predictive value) but the positive predictive value for lethal outcome was only 24%. Among the 15 patients with lethal outcome, nine patients died from septic shock and six patients from severe head injury. The median initial PCT concentration (quartiles) of the septic group was 4.25 (2.05–8.3) ng/ml and that of the head injury group was 1.98 (0.63–3.94) ng/ml (*P *= 0.49). The median ISS were 41 and 50, respectively. In contrast, CRP concentrations during the first week showed no differences between survivors and nonsurvivors.

### Role of infection

In the present study, infection was suspected in 49 of the 90 patients and was proven in 40 patients during the 21-day observation period. Positive microbial findings were derived from pneumonia in 31 cases, from positive blood cultures in 10 patients, from colitis in 10 patients, and from wound and fungal infections in four patients. Five patients had urinary tract infections (multiple microbiological findings). On average, infections occurred 6 ± 3 days (mean ± standard deviation) after the trauma. Initial PCT levels (days 1 and 2 after trauma) were significantly higher in patients who subsequently developed infections: 2.69 ng/ml versus 0.54 ng/ml (median, *P *< 0.001) for suspected infection versus no suspected infection, and 3.01 ng/ml versus 0.57 ng/ml for proven infection versus no proven infection (*P *< 0.001). Initial CRP concentrations (days 1–3) did not significantly differ in patients developing a proven infection (109 mg/l versus 136 mg/l, *P *= 0.028; not significant according to multiple comparisons) or in patients in whom infection was suspected (109 mg/l versus 122 mg/l, *P *= 0.163; not significant according to multiple comparisons). For a PCT value ≥1 ng/ml the odds ratio for the development of an infection was 6.1 (95% confidence interval, 2.4–15.7).

The duration of treatment on the ICU was also different in patients with diagnosis of infection as compared with in those without. On average, patients without an infection were treated in the ICU for 6 ± 4 days (mean ± standard deviation), as compared with 20 ± 12 days for those who had an infection (*P *< 0.05). Among patients without an infection only 16 out of 50 patients (30%) were treated on the ICU for more than 7 days, as compared with 38 out of 40 patients (95%) in whom an infection had developed. Similarly, PCT concentrations on day 7 were significantly higher in patients who had an infection, compared with those without an infection: 0.64 ng/ml (median, quartiles 0.1–8.35) versus <0.3 ng/ml (median, quartiles 0.1–0.57) (*P *< 0.001, Mann–Whitney *U *test). CRP concentrations did not significantly differ at this time point (132 mg/l and 90 mg/l, *P *= 0.052).

### Development of sepsis

**Figure 5:**
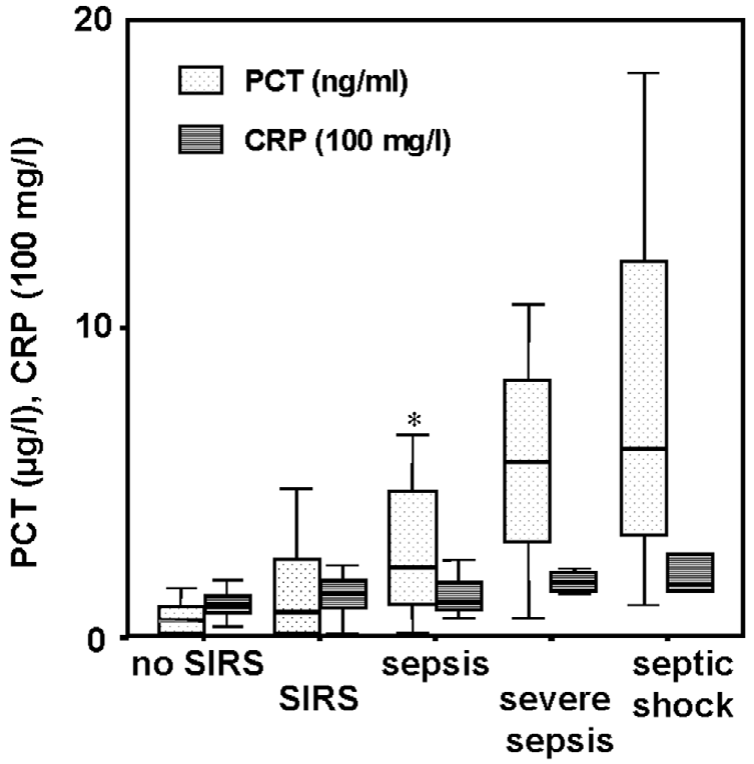
Initial serum concentrations of procalcitonin (PCT) (0–48 hours) and C-reactive protein (CRP) (0–72 hours) after mechanical trauma, and the development of septic complications during a 21-day follow-up. SIRS, systemic inflammatory response syndrome.

**Figure 6:**
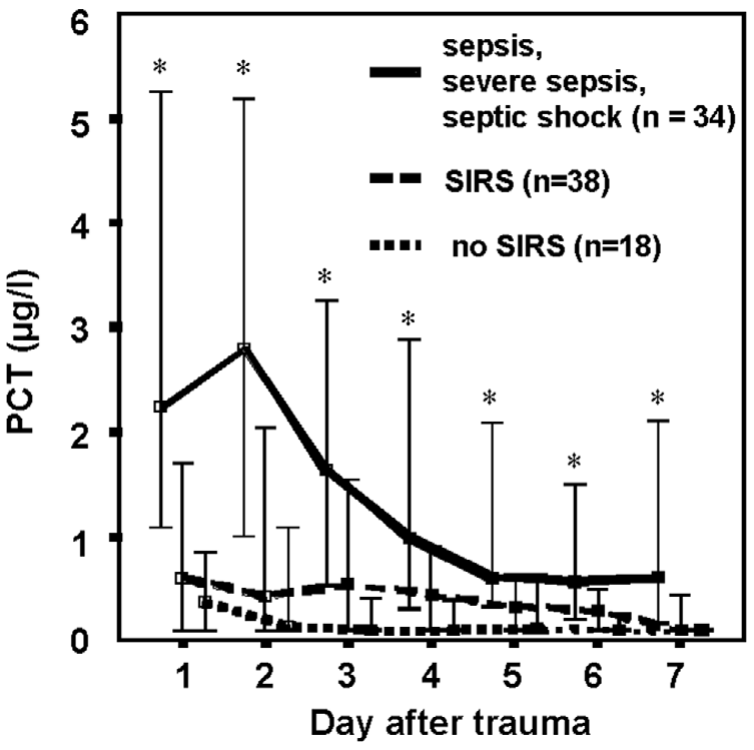
Course of procalcitonin (PCT) in patients who developed various stages of sepsis, systemic inflammatory response syndrome (SIRS), or no sepsis (median and quartiles).

Sepsis, severe sepsis, or septic shock was also more frequently diagnosed during the observation period in patients who initially developed higher PCT levels. On the contrary, the level of the initial CRP concentration was not related to this diagnosis (Figure 5). For example, the initial PCT median (quartiles) concentration in patients who did not develop systemic inflammatory response syndrome (SIRS) or sepsis during their whole course was 0.53 ng/ml (<0.3 to 0.98 ng/ml), compared with those who did develop SIRS (0.77 ng/ml, <0.3 to 2.53; not significant), sepsis (2.21 ng/ml, 1.03–5.16; *P *= 0.003), severe sepsis (5.68 ng/ml, 1.82–9.56; *P *< 0.005) or septic shock (6.06 ng/ml, 2.69–13.4; *P *< 0.005). PCT levels of patients who had either SIRS or sepsis were not significantly different (*P *= 0.021; not significant for multiple comparisons). PCT concentrations remained elevated in patients with sepsis, severe sepsis, or septic shock, but rapidly dropped back to near-normal values in patients without sepsis (Figure 6).

The odds ratio for the development of sepsis/severe sepsis or septic shock was increased for PCT (≥1 ng/ml), for ISS (≥20), and for SOFA score (≥12), but not for the other parameters (*P *< 0.002, chi-square test) (Table 3).

### Development of complications: organ dysfunction

Although high PCT levels related to a higher frequency of developing sepsis, the initial PCT and CRP concentrations did not correlate with the severity of organ dysfunction, measured as maximum SOFA score values during the study period (*r *= 0.438, *P *< 0.01; not significant for multiple comparisons). Moreover, PCT levels measured daily only weakly correlated with the corresponding SOFA score at this time point (*r *= 0.50–0.73 from day 1 to day 14, *P *< 0.05). Similarly, the initial PCT values did not correlate with the APACHE II score, measured 1 day after the trauma had occurred (*r *= 0.395, *P *= 0.01).

## Discussion

The present study was designed to analyze the amount of and the kinetics of induction of PCT and CRP early after trauma depending on the type and the severity of the trauma and its related complication. PCT and CRP were induced in various amounts in patients with mechanical trauma. Both parameters showed different time courses of induction. CRP induction after trauma showed a uniform response with no significant relation to trauma severity, development of the various stages of sepsis, as well as the duration of ICU treatment and outcome. Furthermore, concentrations were elevated for several days after the trauma. PCT induction was moderate, but differed according to the severity of trauma. Patients with high PCT plasma levels more frequently developed various complications, including the prolongation of treatment in the ICU and a worse outcome. Interestingly, compared with the kinetics seen for CRP, plasma levels of PCT declined more rapidly in patients without complications.

PCT and CRP are increasingly used as markers for the diagnosis of sepsis and infection. However, both parameters are also induced independent from infection (for example following cardiogenic shock, major surgery, or mechanical trauma) [1,3,17,18]. In cardiac surgery, PCT is elevated in patients with pulmonary dysfunction and noninfectious SIRS [2,19-22]. Increased plasma levels are related to circulatory failure, the need for high doses of catecholamines, blood transfusions, and surgical re-interventions [2]. Also, after elective general surgery, high postsurgical plasma levels were related with a higher rate of complications. Patients undergoing colon surgery or aortic surgery more frequently developed infections or insufficiency of the anastomosis when PCT concentrations were increased after surgery [23].

Typically, a cutoff PCT-level of 1.5–2 ng/ml has been described as an indicator of increased risk after various types of elective surgery (for example, colon surgery, aortic surgery, and cardiothoracic surgery) [2,23,24].

To compare the increase of PCT in this study in patients who later developed complications with the data of previous studies, we calculated a cutoff value for the diagnosis of sepsis. Contrary to the previous studies, however, this cutoff value only indicates the risk for the development of such complications during the further course of the disease, but not the actual sensitivity or specificity for the diagnosis of sepsis or the respective complications at this specific time point. Nevertheless, PCT with a cutoff value of 1.5 ng/ml had a specificity of 70% for the diagnosis of sepsis in this study. These data were similar to the results of PCT measurements of a study published previously by Wanner and colleagues. They reported a cutoff value of 1.5 ng/ml PCT on day 1 or day 3 after trauma and a sensitivity and specificity of 75.6% and 77.3%, respectively, for the diagnosis of sepsis as compared with the noninfection-related severe SIRS [3].

Also in our study the amount of PCT and CRP typically induced after accidental trauma did not substantially exceed those concentrations reported previously after elective surgery, especially if major surgical procedures were compared [1,2,7,20-22].

PCT concentrations of approximately 1–1.5 ng/ml are also reported in various studies to be the best cutoff level for the diagnosis of sepsis as compared with SIRS [25-28]. Very high PCT concentrations after trauma therefore indicate a substantially increased risk of complications, including sepsis, organ dysfunction, or lethal outcome, whereas moderately increased PCT concentrations (below 1.5–2 ng/ml) are usually not of major concern.

In the present study, PCT levels were not different in patients who underwent surgical procedures early after trauma as compared with those without surgery. We found a tendency to higher PCT levels following abdominal trauma. This is in agreement with former observations in patients undergoing elective surgical procedures at different regions of the organism, where we found higher PCT and CRP levels after surgery of the intestine (PCT: median, 1.50 ng/ml; 90% percentile, 3.0 ng/ml; CRP: median, 131 mg/l; 90% percentile, 230 mg/l) than after minor and less elective traumatic surgery (PCT: median, 0.38 ng/ml; 90% percentile, 0.73 ng/ml; CRP: median, 61 mg/l; 90% percentile, 181 mg/l) [1].

The cause of the induction of PCT after trauma is not completely understood. Experimental data from both *in vivo *and *in vitro *studies have indicated that not only bacterial endotoxins (lipopolysaccharide), but also various other mediators are capable of inducing PCT. In clinical and experimental studies PCT was induced by tumor necrosis factor alpha, IL-2, IL-6, phytohemagglutinin, and other proinflammatory stimuli [29-31]. However, recent *in vitro *experiments provide further data that better explain the trauma-related and infection-related induction and function of PCT. The induction of and action of PCT is a multifactorial process, which involves different cell–cell interactions, a time-dependent pattern of cellular activation, and different cellular behavior when cells are native or pre-exposed to proinflammatory stimuli. In short, only adherent monocytes produce a significant amount of PCT. Circulating monocytic cells do not produce a quantitative amount of PCT. This is the first key for the understanding of PCT induction after trauma, since the expression of adhesion molecules and receptors also occurs in traumatized tissue.

Once induced, PCT acts as a chemokine in this area, attracting further monocytes; however, this property is lost after a few hours, when cells are preincubated or are in contact with PCT, thus limiting the local action to the acute phase response [32]. Furthermore, production of PCT in adherent monocytes is also limited to a period of only a few hours. Later on, parenchymatous cells are a major source for PCT during sepsis [33], but these cells (as investigated presently only for adipocytes) produce major amounts of PCT only when they become directly in contact with activated monocytes [34]. This is another key for the understanding of PCT induction after trauma.

Should systemic inflammation, sepsis, or organ dysfunction occur, there is a continuous and systemic stimulus for the production of PCT, whereas the trauma-related local induction of PCT soon declines, if the inflammation in the traumatized tissue disappears. Consequently, local tissue injury in combination with the activation of the immune system is a major cause for the induction of PCT after trauma. During a systemic inflammatory response such as sepsis there exist additional major sources for PCT (for example, the liver). Increased levels of PCT, compared with lower circulating plasma levels, have been measured in hepatic venous blood [35], and a significant PCT production was absent in an anhepatic baboon during endotoxin shock [36].

Other complications of trauma that are associated with severe tissue trauma (for example, malperfusion due to hemorrhagic shock or trauma-associated blood loss) and transfusion of allogenous blood cells may also contribute to the induction of PCT and may promote the development of organ dysfunction and poor outcome in these patients. PCT induction occurred during hemorrhagic shock in a baboon shock model, but it was weaker than PCT induction during endotoxin shock [37].

The biological action of PCT obviously has a significant influence on the course of the systemic inflammation and sepsis. The mortality rate of hamsters decreased when calcitonin precursors where neutralized [38], and the mean arterial pressure and urine output increased after the infusion of neutralizing antibodies against calcitonin precursor molecules in a porcine model [39]. *In vitro*, PCT augmented the induction of inducible nitric oxide synthase in cultured smooth muscle cells preincubated with proinflammatory stimuli [40]. A negative effect of high PCT levels in multiple-trauma patients in the further course of inflammation and the development of complications such as organ dysfunction cannot be excluded at present.

## Conclusion

PCT and CRP levels measured early after trauma increase in patients with severe trauma. In addition to previous studies, we have analyzed the various types of trauma and have compared PCT with CRP and various clinical score systems. Unlike CRP, PCT values typically declined rapidly after trauma. The rapid decline of the trauma-induced response of PCT towards its normal range compared with the long-lasting increase of CRP promises an earlier diagnostic use of PCT as a marker of sepsis and infection than of CRP, since sepsis and infection can be diagnosed with high specificity only if there is no or only minor unspecific induction [26,41]. Our data demonstrate that the CRP level in trauma patients is not a valid parameter to gather more information about the severity of systemic inflammation, complications, and prognosis of the patient. PCT and clinical score systems are equally superior to CRP for risk-stratification of the patient. However, since PCT can additionally be used as a marker for the diagnosis of sepsis soon after the initial trauma-related increase has again declined, the measurement of PCT is the superior parameter of choice to diagnose sepsis in these patients. On the contrary, the diagnostic use of CRP after trauma is limited since significant induction occurs in almost all patients, and its kinetics are slow.

## Key messages

• 	The inflammation-related markers PCT and CRP increase early after trauma.

• 	The increase for PCT, but not for CRP, is somewhat related to the severity of trauma but remains within moderate levels (<1 to 2 ng/ml), whereas CRP levels increase during even minor trauma (>100 mg/l).

• 	The kinetics of PCT increase and its decline after trauma is faster than that seen for CRP (peak levels of PCT occur most often on day 1 or day 2 after trauma, and that of CRP on days 2, 3 or 4 after trauma).

• 	PCT levels, but not CRP levels, elevated early after trauma are related to a higher rate of posttraumatic complications (for example, sepsis, severe sepsis, septic shock), more frequent infection, a worse outcome, and longer duration of treatment on the ICU.

• 	The more rapid decline of PCT levels towards its normal range allows an earlier use of this marker for its usual indications than CRP, whose levels are elevated for a long time.

## Abbreviations

APACHE II = Acute Physiology and Chronic Health Evaluation II; CRP = C-reactive protein; ICU = intensive care unit; IL = interleukin; ISS = Injury Severity Score; PCT = procalcitonin; SIRS = systemic inflammatory response syndrome; SOFA = Sepsis-related Organ Failure Assessment.

## Competing interests

MM received support for holding lectures on inflammation markers from BRAHMS Diagnostica AG, Germany.

## Authors' contributions

MM developed the study design and coordinated its implementation. MM and JS participated in interpretation/discussion of results and drafted and revised the manuscript. MM and HA were responsible for patient recruitment as well as data collection and they carried out the statistical analysis. All authors read and approved the final manuscript.

## Figures and Tables

**Table 1:** Patient characteristics and infection markers early after minor or major severe mechanical trauma

	Injury Severity Score <20	Injury Severity Score ≥20	*P*, Mann–Whitney *U *test
Group characteristic	31 patients; Injury Severity Score, 14 ± 4	59 patients; Injury Severity Score, 30 ± 9	
Age (mean ± standard deviation)	28 ± 14	31 ± 17	>0.9
Gender (male/female)	21/10	40/19	>0.9
Lethal outcome (*n*)	0	15	<0.01
Initial procalcitonin (0–48 hours) (median, quartiles) (ng/ml)	0.52 (<0.3 to 1.1)	2.43 (0.79–5.16)	<0.01
Initial C-reactive protein (0–72 hours) (median, quartiles) (mg/l)	126 (65–151)	120 (82–169)	<0.17

**Table 2:** Induction of procalcitonin (PCT) and C-reactive protein (CRP) according to the region of injury, defined by the Injury Severity Score

Region of injury	Number of patients with injury in this region		Median (10th–90th percentiles)		*P *(exact significance, two-tailed)
				
			Injury in the respective region	No injury in the respective region	
Head	67/90	PCT(ng/ml)	1.3 (0.1–6.68)	0.9 (0.1–5.2)	0.562
		CRP(mg/l)	115 (37–197)	121 (54–220)	0.762
Face	47/90	PCT(ng/ml)	1.0 (0.1–7.89)	1.4 (0.1–5.4)	0.976
		CRP(mg/l)	109 (19–206)	121 (56–193)	0.621
Thorax	72/90	PCT(ng/ml)	1.31 (0.1–6.4)	0.75 (0.1–5.0)	0.121
		CRP(mg/l)	115 (32–194)	136 (72–222)	0.189
Abdomen	44/90	PCT(ng/ml)	2.32 (0.1–12)	0.67 (0.1–5.0)	0.004
		CRP(mg/l)	136 (41–217)	107 (36–173)	0.080
Extremities	71/90	PCT(ng/ml)	1.3 (0.1–6.46)	0.85 (0.1–5.0)	0.573
		CRP(mg/l)	120 (48–186)	97 (11–226)	0.358
Surface	45/90	PCT(ng/ml)	1.03 (0.1–6.0)	1.56 (0.1–7.0)	0.792
		CRP(mg/l)	120 (43–178)	120 (14–220)	0.584

**Table 3:** Sensitivity and specificity for the prediction of various complications in multiple-trauma patients

Parameter (cutoff value)	Predicted risk				
	
	Outcome	Infection	Sepsis/severe sepsis/septic shock	SOFA score ≥12 within 21 days	ICU stay >7 days
	
	AUC (odds ratio, 95% CI) (*P *< 0.002), chi-square test	Sensitivity/specificity (cutoff value)	AUC (odds ratio, 95% CI) (*P *< 0.001)	Sensitivity/specificity (cutoff value)	AUC (odds ratio, 95% CI) (*P *< 0.002)
Initial procalcitonin (≥1 ng/ml)	0.732 (-, not increased)	93%/41% (0.62 ng/ml)	0.805 (6.1, 2.5–15.7)	90%/48% (0.53 ng/ml)	0.798 (7.8, 2.8–21.9)
		80%/59% (1.31 ng/ml)		80%/64% (0.97 ng/ml)	
		73%/60% (1.37 ng/ml)		70%/74% (1.36 ng/ml)	
Initial C-reactive protein (>100 mg/l)	0.564 (-, not increased)	87%/37% (105 mg/l)	0.674 (-, not increased)	90%/30% (76 mg/l)	0.639 (-, not increased)
		80%/41% (111 mg/l)		80%/64% (104 mg/l)	
		73%/45% (122 mg/l)		70%/52% (121 mg/l)	
Injury Severity Score (≥20)	0.869 (1.3, 1.1–1.6)	100%/61% (26.0)	0.760 (10.6, 3.3–34.1)	90%/54% (20.0)	0.721 (7.0, 2.2–22.4)
		80%/77% (31.5)		88%/54% (21.5)	
		73%/80% (34.0)		73%/70% (26.0)	
Initial SOFA score (≥12)	0.864 (11.6, 3.3–40.6)	87%/68% (10.5)	0.805 (6.0, 2.0–18.4)	95%/42% (7.5)	0.788 (4.9, 1.7–13.9)
		73%/76% (11.5)		82%/70% (9.5)	
		67%/85% (12.5)		65%/78% (10.5)	
APACHE II score (≥20)	0.783 (13.1, 3.5–48.9)	93%/39% (13.5)	0.685 (-, not increased)	90%/40% (12.5)	0.678 (-, not increased)
		87%/48% (14.5)		82%/46% (13.5)	
		73%/61% (15.5)		57%/66% (15.5)	

APACHE, Acute Physiology and Chronic Health Evaluation; AUC, area under the curve of the receiver operating characteristic; CI, confidence interval; SOFA, Sepsis-related Organ Failure Assessment. AUCs were not significantly different among parameters, when compared with the statistical method of Hanley and McNeil (*z *< 1.88) for outcome [16].
